# Determinants of maternity waiting home utilization among women who gave birth in public health facilities in the Gedeo Zone, southern Ethiopia: an unmatched case–control study

**DOI:** 10.3389/fgwh.2023.1170843

**Published:** 2023-08-15

**Authors:** Dawit Tilahun, Mohammed Feyisso Shaka, Moges Mareg Belay

**Affiliations:** Department of Reproductive Health, Dilla University College of Health Sciences and Medicine, Dilla, Ethiopia

**Keywords:** maternal waiting home, utilization, case–control study, southern Ethiopia, determinants

## Abstract

**Background:**

Maternal mortality remains unacceptably high in Ethiopia, although most of its causes are preventable. One way of tackling this problem is by establishing a maternal waiting home (MWH) close to a health facility. Although the benefits of an MWH have been well-documented, the determinants of its use have not been well-studied. This study aims to identify the determinants of utilization of an MWH among women who gave birth in public health facilities in the Gedeo Zone, southern Ethiopia.

**Methods:**

A facility-based unmatched case–control study was conducted between January 2020 and February 2020) among 129 patients belonging to the case group and 257 belonging to the control group. The data were entered into the Epi-Data version 3.1 and exported to the SPSS version 20 statistical package for analysis. Descriptive statistics such as frequency, means, and standard deviations were computed. The association between variables was checked using logistic regression analysis, and odds ratios (ORs) with 95% confidence interval (CI) were used to determine the strength of this association. A *p*-value of < 0.05 was used as a cutoff point to measure statistical significance.

**Result:**

A total of 378 respondents (126 cases and 252 controls) were included in the study, successfully achieving a response rate of 97.9%. The mean age of the participants was 27.4 (±5.6 SD) years, which was 28.4 (±5.5 SD) years for case group patients and 26.9 (±5.69 SD) years for control group patients. The educational status of women [adjusted odds ratio (AOR): 8.49, 95% CI: 2.91–24.7], travel time (AOR: 2.92, 95% CI:1.41–4.67), antenatal care visits (AOR: 3.54, 95% CI: 1.33–9.38), those having more than two children under the age of 5 years (AOR: 0.12, 95% CI: 0.06–0.26), those with a history of complications in previous childbirths (AOR: 4.52 95% CI: 2.41–8.47), previous place of delivery (AOR: 6.30, 95% CI: 2.71–14.78), and a lack of awareness (AOR: 5.8, 95% CI: 2.23–15.2) were all significantly associated with the utilization of an MWH.

**Conclusion:**

Educational status, antenatal care follow-up, number of children under 5 years old in the household, previous place of delivery, lack of awareness regarding maternal waiting home service, and travel time were all determinants of MWH use. This implies that interventions focusing on promoting antenatal care visits, institutional delivery, and raising awareness of the benefits of MWHs are important for improving their rate of utilization.

## Introduction

Maternity waiting homes (MWHs) are defined as “residential facilities located within or close to the health facility to accommodate women in their final weeks of pregnancy” (1, 2). The MWH strategy is used to reduce maternal and perinatal mortality by improving access to skilled birth attendance and emergency obstetric care, particularly for women in rural and remote areas.

In Ethiopia, the first set of MWHs was opened in Attat Hospital in the year 1976 because of the difficulty involved in pregnant women reaching hospitals and health centers and the high number of obstetric emergencies (1, 2). However, MWHs have been in existence in Ethiopia for more than 45 years, and the expansion to lower-level health facilities is a very recent initiative in the country (3).

Maternal mortality is unacceptably high in Ethiopia. Globally, approximately 295,000 women died during and after pregnancy and childbirth in the year 2017. The vast majority of these deaths (94%) occurred in low-resource settings; however, more than 80% of these deaths could have been prevented (4). In Ethiopia, the maternal mortality ratio dropped from 676 to 412 per 100,000 live births between 2011 and 2016, but Ethiopia was still among 15 countries considered “very high alert countries” or countries contributing to high maternal mortality, according to the Fragile States Index report in 2017 (4).

Most of these deaths occur because of known and direct causes that are mostly preventable by the use of maternal healthcare services (4–7). In Ethiopia, there is a 92% reduction in maternal deaths among MWH users when compared with non-users and there is 73% less occurrence of stillbirth among users (8). On the other hand, admission to an MWH shows an improvement rate of 27.5% in overall maternal and perinatal obstetric outcomes (9). This shows that MWH use will play a great role in achieving sustainable development goals (SDGs) for 2030 with regard to reducing maternal mortality (70 per 100,000 live births) as well as reducing neonatal mortality to at least as low as 12 per 1,000 live births (10, 11). Although many studies reveal that the use of MWHs lowers the risk of maternal and perinatal deaths (12–14), the utilization levels of MWHs have generally been reported to be low: MWH use in Kenya is only 10%, and it is only 33% in Zimbabwe (14, 15). The utilization of existing MWH facilities varies substantially across different parts of Ethiopia (6, 15).

According to the 2016 emergency obstetric and neonatal care (EmONC) assessment of 3,804 health facilities, even if the average number of MWH beds was seven, on average, it was found that only two women had used an MWH (1, 16). The most frequently mentioned factors that prevent women from utilizing MWHs are a lack of family and community support (13), poor awareness of the presence or benefits of an MWH, educational status, marital status, travel time (17, 18), prior complications, refusal from patients’ husbands (4, 16), a lack of awareness about the risks of pregnancy, and a history of previous cesarean delivery (13, 19). Globally, as well as in Ethiopia, research has been done on the positive effects of MWHs on maternal and perinatal health outcomes. The Federal Ministry of Health of Ethiopia has designed a policy and strategy that promotes the implementation of MWHs (16). Despite this effort, the utilization of existing maternal waiting homes varies in different parts of the country (10, 20, 21). Different studies have identified the determinants of MWH utilization, but they have failed to use a strong study design. Since no study has been conducted on MWH utilization in Gedeo zone, the present study aims to identify the determinants of MWH utilization among mothers who gave birth in public health facilities in the Gedeo Zone in southern Ethiopia.

## Methods and materials

### Study area and period

The study was conducted in public health facilities (health centers) in the Gedeo Zone between 11 January 2020 and 25 February 2020. This zone is located in the southern part of Ethiopia, 371 km from Addis Ababa, the capital city of Ethiopia, and 96 km from Hawassa City, the capital of the South Nation Nationality and People Regional (SNNPR). The Gedeo Zone is bordered by the Boreana Zone of the Oromia regional state in the south and west, the Sidama Zone of the SNNP region in the north, and the Guji Zone of the Oromia regional state in the east. The Gedeo Zone covers a total area of 5,890.2 km^2^ and comprises eight districts and four administrative towns. Based on the HMIS Data 2019 report, the projected total population of the zone is 1,226,168, according to the 2019 Ethiopian fiscal year (EFY), of which 625,345 are women and 600,823 are men. Currently, this zone has one referral hospital, three primary hospitals, and 38 health centers; all of the health centers have MWH facilities; there are 146 health posts, 12 private clinics, and four NGO clinics.

### Study design

A facility-based unmatched case–control study design was employed.

### Source population

All women who gave birth after being admitted to an MWH in a health facility in the Gedeo Zone were the source population for the case group. All women who gave birth directly without gaining admission to the health facilities in the zone were the source population for the control group.

### Study population

The study population for the case group was women who gave birth after being admitted to an MWH in a selected health facility. The study population for the control group was women who gave birth in health facilities directly without getting admitted to an MWH in a selected health facility.

### Inclusion criteria

All women who gave birth in a health facility after being admitted to an MWH during the study period and residing in the Gedeo Zone were included in the case group. All women who gave birth directly without being admitted to an MWH during the study period and residing in the Gedeo Zone were included in the control group.

### Exclusion criteria

Women who were critically ill and unable to participate in the data collection process were excluded.

### Sample size determination

The sample size was determined by using EPI INFO version 7.2.3.1 statistical software package with the following considerations: 20.5% of controls exposed for determining the educational level of the husband, 95% confidence level, power = 80, and odds ratio (OR) = 2.1, which provided the maximum sample size (see Table 1). The evidence was collected from another facility-based study (9, 15). After adding 10% for determining the non-response rate, 129 cases and 257 controls (a total sample size of 386) were included in the study.

**Table 1:** Sociodemographic characteristics of the respondents among women who gave birth in a public health facility in Gedeo Zone, southern Ethiopia, 2020.

Variables	Cases (*n* = 126)	Controls (*n* = 252)
	Frequency (%)	Frequency (%)
Age of respondents (years)		
<20	8 (6.3%)	16 (6.2)
20–34	92 (73.1%)	201 (79.9%)
≥35	26 (20.6)	35 (13.9%)
Marital status		
Single	10 (7.9%)	17 (6.7%)
Married	102 (81.1%)	193 (76.6%)
Divorced or widowed	14 (11.0%)	42 (16.7%)
Educational status		
No formal education	25 (19.8%)	111 (44%)
Primary school (1–8)	35 (27.8%)	75 (29.8%)
Secondary school (9–12)	66 (52.4%)	66 (26.2%)
Educational status of husband		
No formal education	57 (45.2%)	94 (37.6%)
Primary education	35 (27.8%)	91 (36.4%)
Secondary education and above	34 (27%)	65 (26%)
Occupational status		
Housewife	68 (53.9%)	142 (56.8%)
Government employee	11 (8.7%)	25 (10.0%)
Merchant	33 (26.2%)	16 (6.4%)
Student	9 (7.2%)	34 (13.6%)
Other^a^	5 (4%)	33 (13.2%)
Occupational status of husband		
Farmer	59 (46.8%)	117 (46.4%)
Government employee	33 (26.2%)	24 (9.5%)
Daily labor/wage labor	25 (19.8%)	85 (33.7%)
Other^b^	9 (7.2%)	26 (10.4%)
Religion		
Protestant	67 (53.2%)	134 (52.3%)
Orthodox	35 (27.8%)	75 (29.8)
Catholic	16 (12.7%)	27 (10.7%)
Muslim	8 (6.3%)	16 (6.3%)
Wealth status		
Wealthy	17 (13.5%)	33 (13.1%)
Medium	58 (46%)	75 (29.8%)
Poor	51 (40.5%)	144 (57.1%)
Travel time to nearest MWH (min)		
<60	41 (32.5%)	195 (77.4%)
>60	85 (67.5%)	57 (22.6%)

^a^ Farmer and daily labor.

^b^ Merchant and student.

### Sampling procedure

**Figure 1:**
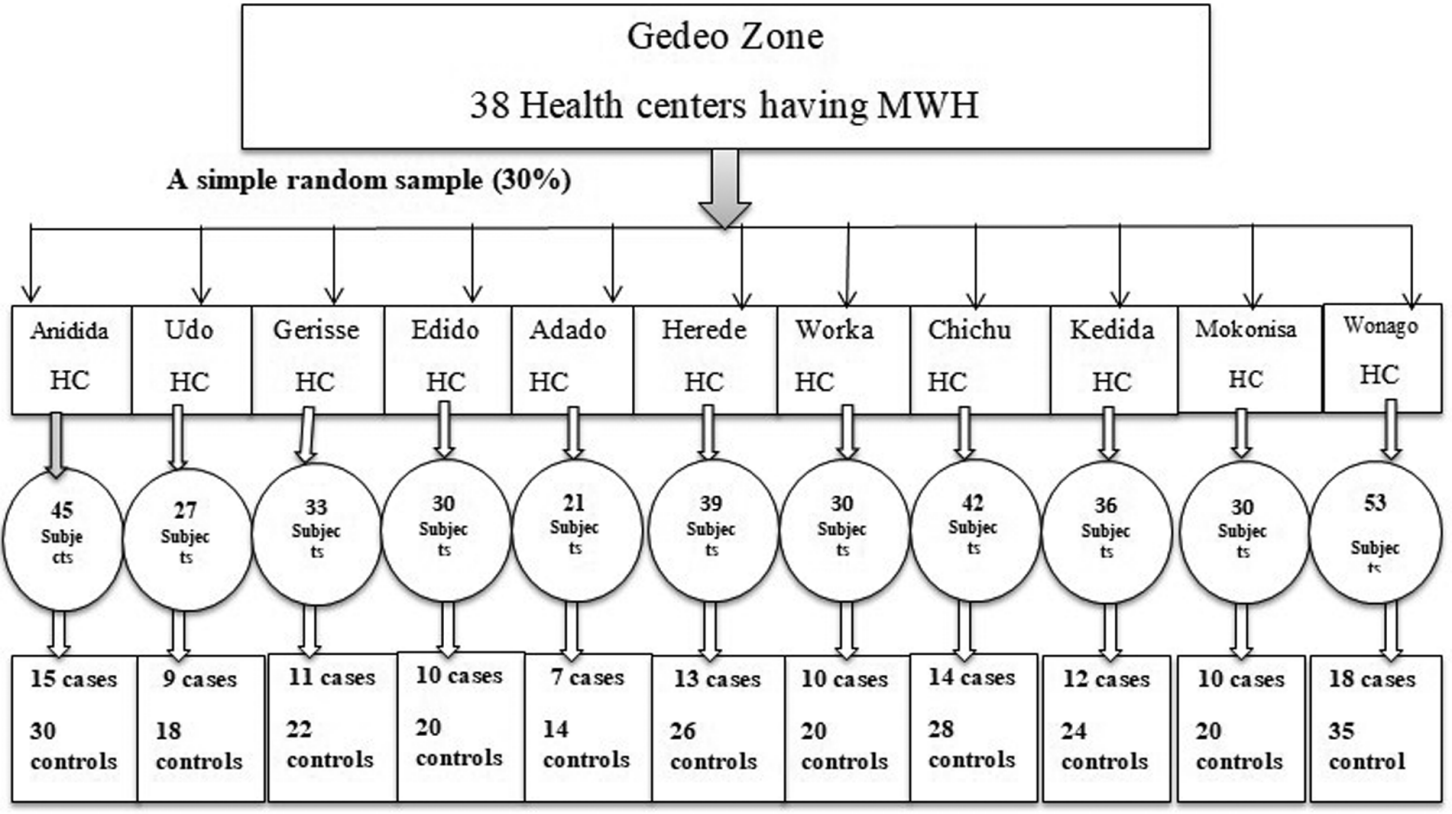
Sampling procedure.

A total of 38 health centers have an MWH in the Gedeo Zone, and 30% (11 health centers) were selected using a simple random sampling technique by the lottery method. Among the health centers that were selected, the monthly patient flow of each facility, for both the case and the control groups, was enumerated from the record of health centers [2 months of the previous year (January and February), and this duration was similar to that of the study period]. Then, based on the monthly patient flow, the sample size was proportionally allocated for all health centers. A systematic sampling technique was used to recruit the study unit while they were in the postnatal care (PNC) room. The cases were recruited at every interval of 1 (323/129), whereas the controls were recruited using an interval of 3 (779/257) consecutively. Finally, both the cases and the controls were selected, and for each case, two consecutive controls were selected. The identified women were informed of the objectives of the study and were invited to participate (Figure 1).

### Data collection procedure

The data were collected through face-to-face interviews with 11 midwives who worked at the labor wards and who were fluent in speaking the Gedeuffa language. They were recruited as data collectors, and two public health officers were assigned as supervisors. The interviews were conducted confidentially after securing written informed consent. Close supervision was done by principal investigators and supervisors throughout the data collection period. The collected data were checked for completeness and consistency of responses on a daily basis.

### Data quality control

To assure data quality, great emphasis was placed on designing the data collection instruments. Two days of training were given for data collectors and supervisors on how to use the study instrument, on how to use the consent form, on the data collection procedure, and on the purpose of the study. Before starting the actual survey, the questionnaire was pretested on a 5% total sample size (seven cases and 14 controls, with a total of 21 subjects) in Dara district. After analyzing the data from the pretest, an amendment was made, and some unclear questions were rephrased. Data were checked for completeness before being entered into the Epi-Data and for validity (selection of each case and control following randomization). The reliability of data was assured throughout the data collection process. Regular meetings were held between the data collectors and the principal investigators in which problematic issues arising from interviews were discussed, and if mistakes were found, steps were taken to correct them and resolve any other problems that cropped up during data collection.

### Dependent variable

The dependent variable was MWH utilization.

### Independent variables

• Sociodemographic variables: Age, maternal education, religion, educational level of the husband, maternal occupation, occupation status of the husband, marital status, and wealth index.
• Obstetric and gynecologic history–related variables: ANC visit, complications in previous birth, cesarean section history, parity, number of children in the household, and the outcome of recent pregnancy.
• Health service–related variables and other variables: Average waiting time, place of delivery, decision- maker, husband support, awareness of MWHs and transportation access, and travel time.

### Term definitions of the study variable

Travel time: travel time to the nearest MWH (≥1 h = inaccessible, <1 h = accessible) (6).
Cases (MWH users): women who gave birth after being admitted to an MWH irrespective of the risk (22).
Controls (non-MWH users): women who gave birth in the health facilities directly without admission (3).
Awareness related to MWH service: Those respondents who scored the mean score and above on the awareness-related questions were considered “having awareness” and those who scored below the mean were considered “not having awareness”.

### Data processing and analysis

The data were entered using Epi-Data version 3.1 and then exported and analyzed by using SPSS version 20. Descriptive statistics such as frequency distribution cross-tabulation and some measures of central tendency and variability (mean and standard deviation) were computed to describe the major variables of the study. The odds ratio and *p*-value were computed to see whether any relationship exists between the exposure and the outcome variables. Factors with a *p*-value of < 0.25 in the bivariate analysis were entered into the multivariate regression analysis for further analysis and for controlling potential confounders. Before performing the multivariate analysis, independent variables were checked for the multicollinearity effect using the variance inflation factor (VIF) between variables of less than 5. In the multivariate logistic regression analysis, *p* < 0.05 was used as a cutoff point to measure the association of statistical significance. The fitness of the model was checked using the Hosmer–Lemeshow test, which confirmed that the model was a good fit (*p* = 0.72).

## Results

### Sociodemographic characteristics of the participants

A total of 378 participants (126 cases and 252 controls) were included in this study, and the response rate was 97.9% (97.6% for cases and 98% for controls). The mean age of the participants was 27.4 years (SD ± 5.6) with 28.4 ± 5.5 years for cases and 26.9 ± 5.69 years for controls.

More than half of the case group patients (67, 53.2%) and control group patients (134, 52.3%) were followers of the protestant religion, followed by followers of the Orthodox Church (35, 27.8%) cases and (75, 29.8) controls. A total of 41 (32.5%) of the cases and 195 (77.4%) of the controls reported being able to reach a health center providing obstetrical services in less than 60 min (Table 1).

### Obstetric and gynecologic characteristics of the respondents

A total of 126 case group patients included in this study (60.3%) had underwent ANC follow-up, and 23.7% of the participants had attended four or more ANC follow-ups; more than half of the case group patients (56.6%) had visited health posts, and the remaining 43.6% had visited health centers for ANC follow-up (Table 2).

**Table 2:** Obstetrics and gynecologic characteristics of the respondents among women who gave birth in a public health facility in Gedeo Zone, southern Ethiopia, 2020*.*

Variables	Cases (*n* = 126)	Controls (*n* = 252)
	Frequency (%)	Frequency (%)
ANC follow-up		
Yes	76 (60.3%)	66 (26.2%)
No	50 (39.7%)	186 (73.8%)
Frequency of ANC visit		
Once	20 (26.3%)	9 (13.6%)
Twice	5 (6.5%)	32 (48.5%)
Three times	33 (43.4%)	17 (25.8%)
Four and above times	18 (23.7%)	8 (12.1%)
Place of ANC visit		
Health center	33 (43.4%)	8 (12.1%)
Health post	43 (56.6%)	58 (87.9%)
Awareness of pregnancy complications		
Yes	92 (73%)	109 (43.3%)
No	34 (27%)	143 (56.7%)
Experience of pregnancy-related complications		
Yes	32 (34.8%)	50 (45.9%)
No	60 (65.2%)	59 (54.1%)
History of cesarean section		
Yes	16 (12.6%)	16 (6.3%)
No	110 (87.3%)	236 (93.7%)
Parity		
One child	8 (6.3%)	20 (7.9%)
Two to four children	85 (67.5%)	85 (33.7%)
Five children and above	33 (26.2%)	147 (58.3%)
The outcome of previous pregnancy		
Live birth	113 (89.6%)	241 (95.6%)
Stillbirth	9 (7.1%)	6 (2.3%)
Other^a^	4 (3.1%)	5 (2%)

^a^ Abortions and live birth that died within 1 day.

### Health service utilization characteristics and awareness of MWHs of the respondents

With regard to access to transportation, two-thirds of the cases (84, 66.7%) and above one-third of the controls (97, 38.5%) had transportation access (either a car/ambulance or motorbike) to visit an MWH. Women were asked who in their households usually makes decisions about their health, and 58 (46%) case group patients and 63 (25%) control group patients reported that decisions are made jointly, whereas 43 (34.1%) case group patients and 90 (35.7%) control group patients are not involved in any decision-making to visit a health facility—rather such decisions are taken by the husband or partner (Table 3).

**Table 3:** Characteristics of health service utilization of the respondents among women who gave birth in a public health facility in the Gedeo Zone, southern Ethiopia, 2020*.*

Variables	Cases (*n* = 126)	Controls (*n* = 252)
	Frequency (%)	Frequency (%)
Transportation access to MWH		
Yes	84 (66.7%)	97 (38.5%)
No	42 (33.3%)	155 (61.5%)
Husband allows stay in MWH		
Yes	92 (73%)	100 (39.7%)
No	34 (27%)	152 (60.3%)
Awareness of MWH		
Yes	84 (66.7%)	63 (25%)
No	42 (33.3%)	189 (75%)
Husband helps with household work		
Yes	96 (76.2%)	92 (36.5%)
No	30 (23.8%)	160 (63.5%)
Decision-maker		
Respondent	25 (19.8%)	99 (39.3%)
Husband/partner	43 (34.1%)	90 (35.7%)
Jointly	58 (46%)	63 (25%)

With regard to awareness on MWHs, more than half (84, 66.7%) of the case group patients had awareness on maternity waiting services, and the majority of them were aware about the advantages of staying in MWHs. On the other hand, only 61 (24.2%) out of 252 control group patients were aware about the advantages of staying in MWHs.

Among the participants, 25 (29.8%) case group patients and 17 (27.9%) control group patients said staying in MWHs saved the life of the mother, while 17 (20.2%) cases and 15 (24.6%) controls said that staying in MWHs saved the life of the baby. The rest, 34 (40.5%) and 8 (9.5%) of the cases and 15 (24.5%) and 14 (23%) of the controls knew the advantage of staying in a MWH was too close to emergency obstetric care center (EmOC) and to take rest before delivery, respectively.

Twenty-five (19.8%) of the respondents stayed only for 1 week in an MWH, and approximately 34 (27%) stayed for 2 weeks. Nearly two-fifths (34.1%) of the women stayed more than 3 weeks in an MWH, and the remaining 24 (19%) stayed in an MWH for more than 1 month (Table 4).

**Table 4:** Reasons for MWH stay among women who gave birth in a public health facility in the Gedeo Zone, southern Ethiopia, 2020*.*

Reasons for use	Frequency	Percentage
Fear of complication	41	32.50%
HEW referral	8	6.30%
Prior use of MWH	17	13.50%
Previous history of CS	8	6.30%
Need rest before delivery	26	20.60%
Multiple pregnancies	9	7.10%
Living far away from HF	17	13.50%
Total	126	100%

CS, caesarean section; HF, health facility.

Even if women prefer to stay in maternity homes, different factors can influence their decision on MWH use. A total of 35 (27.8%) cases and 210 (83.3%) controls of the respondents reported different factors that prevented them from using MWHs.

### Determinants of maternity waiting for home utilization

In the bivariate analysis, *p*-values less than 0.25 were included in the multivariable logistic regression analysis. After adjustment for possible confounders, the variables found to be independently associated with the outcome variables that have a significant association with MWH use were educational status, travel time, ANC follow-up, number of children under 5 years old in the household, pregnancy-related complications, previous place of delivery, and lack of awareness on MWH services.

Women with a higher level of educational status are 8.4 times more likely to use an MWH than those who have no formal education (AOR =  8.49, 95% CI: 2.91–24.7). Women who travel more than 30 min to access MWHs are 2.57 times more likely to use MWHs than those who travel for shorter durations (AOR = 2.57, 95% CI: 1.41–4.67). ANC visits increase the odds of using an MWH facility: women visiting ANC centers are 3.5 times more likely to use MWHs than those who do not visit such centers (AOR = 3.54, 95% CI: 1.33–9.38). Women who have more than two children under 5 years of age in their household have 88% reduced odds of using an MWH compared with those who have two children or less under 5 years of age (AOR = 0.12, 95% CI: 0.06–0.26). Women who have experienced complications during previous deliveries are 4.52 times more likely to utilize MWHs than those who have not had any complications during previous deliveries (AOR =  4.52, 95% CI: 2.41–8.47). Women who deliver in health institutions are 6.3 times more likely to use MWHs than those who deliver at home (AOR = 6.3, 95% CI: 2.71–14.78). Women who have awareness on the benefits of staying in an MWH are 5.8 times more likely to use the facility than those with lower awareness (AOR = 5.8, 95% CI: 2.23–15.20). Age, educational status of the husband, wealth status, parity, and other factors did not show any significant association with MWH use in this study (Table 5).

**Table 5:** Bivariate and multivariate analysis among women who gave birth in a public health facility in the Gedeo Zone, southern Ethiopia, 2020*.*

Variables	Case (*n* = 126)	Control (*n* = 252)	COR (95% CI)	AOR (95% CI)
	Frequency (%)	Frequency (%)		
Age of respondents (years)				
<20	8 (6.3%)	16 (6.2)	1	1
20–34	92 (73%)	201 (79.8%)	0.915 (0.37–2.21)	0.72 (0.20–2.61)
≥35	26 (20.6)	35 (13.9%)	1.486 (0.55–3.99)	1.86 (0.36–9.42)
Marital status				
Single	10 (7.9%)	17 (6.7%)	1	1
Married	102 (81%)	193 (76.6%)	1.12 (0.46–2.69)	2.26 (0.07–67.11)
Divorced and widowed	14 (11.0%)	42 (16.7%)	0.8 (0.29–2.24)	4.01 (0.06–27.24)
Educational status				
No formal education	25 (9.8%)	111 (44%)	1	1
Primary school (1–8)	35 (27.8%)	75 (29.8%)	2.07 (1.14–3.34)*	1.24 (0.32–4.73)
Secondary and above	66 (52.4%)	66 (26.2%)	4.44 (2.5–7.76)***	8.49 (2.9–24.7)***
Educational status of husband				
No formal education	57 (45.2%)	94 (37.3%)	1	1
Primary education	35 (27.8%)	91 (36.5%)	0.62 (0.37–1.04)	0.62 (0.37–1.04)
Secondary and above	34 (27%)	65 (25.7%)	0.85 (0.50–1.44)	0.85 (0.50–1.44)
Wealth status				
Wealthy	17 (13.5%)	33 (13.1%)	2.18 (1.36–3.4)***	2.19 (0.71–6.71)
Medium	58 (46%)	75 (29.8%)	1.45 (0.74–2.8)	0.91 (0.39–2.14)
Poor	51 (40.5%)	144 (57.1%)	1	1
Travel time to nearest MWH (min)				
<60	41 (32.5%)	195 (77.4%)	1	1
>60	85 (67.5%)	57 (22.6%)	7.09 (4.4–11.4)***	2.57 (1.4–4.61)*
ANC follow-up				
Yes	76 (60.3%)	66 (26.2%)	4.28 (2.7–6.74)***	3.54 (1.33–9.38)**
No	50 (39.7%)	186 (73.8%)	1	1
Experience of pregnancy-related complications				
Yes	92 (73%)	109 (43.3%)	3.55 (2.2–5.65)***	4.52 (2.41–8.47)***
No	34 (27%)	143 (56.7%)	1	1
History of CS				
Yes	8 (12.6%)	16 (6.3%)	1 (0.41–2.44)	3.51 (0.15–79.31)
No	118 (87.3%)	236 (93.7%)	1	1
Parity				
One child	10 (6.3%)	20 (7.9%)	1	1
Two to four children	85 (67.5%)	85 (33.7%)	1.78 (0.72–4.39)	1.49 (0.51–3.45)
Five children and above	33 (26.2%)	147 (58.3%)	4.45 (2.74–7.2)***	1.51 (0.84–2.71)
Number of children <5 years in household				
One	59 (46.8%)	33 (13.1%)	1	1
Two	41 (32.5%)	77 (30.6%)	0.29 (0.16–0.53)**	0.05 (0.02–0.12)***
More than two	26 (20.6%)	142 (56.3%)	0.12 (.05–0.18)***	0.12 (0.05–0.26)***
Transportation access				
Yes	84 (66.7%)	97 (38.5%)	3.19 (2.04–5.0)***	1.2 (0.51–2.8)
No	42 (33.3%)	155 (61.5%)	1	1
Husband allows stay in MWH				
Yes	92 (73%)	100 (39.7%)	4.11 (2.57–6.5)***	1
No	34 (27%)	152 (60.3%	1	1.94 (0.9–4.18)
Awareness of MWH				
Yes	84 (66.7%)	63 (25%)	6 (3.76–9.57)***	1
No	42 (33.3%)	189 (75%)	1	5.8 (2.23–15.2)***
Decision-maker				
Respondent	25 (19.8%)	99 (39.3%)	1	1
Husband/partner	43 (34.1%)	90 (35.7%)	1.13 (0.49–2.59)	1.55 (0.49–2.59)
Jointly	58 (46%)	63 (25%)	0.75 (0.28–1.99)	0.8 (0.33–1.97)
History of place of delivery				
Health facility	109 (86.5%)	121 (48%)	6.94 (3.9–12.2)***	6.3 (2.71–14.7)***
Home	17 (13.5%)	131 (52%)	1	1

COR, crude odd ratio.

* *p* < 0.05.

** *p* < 0.01.

*** *p* < 0.001.

## Discussion

This study found that educational status, ANC follow-up, number of children under 5 years old in the household, pregnancy-related complications, previous place of delivery, lack of awareness on MWH service, and travel time showed a significant association with MWH use.

The educational attainments of women were found to be a very significant factor in the use of MWHs. Unlike women with no formal education, those with secondary education and above were 8.4 times more likely to use an MWH. Other studies conducted in Sudan (17), Butajira (23), and the Gurage Zone also found similar results (6). A possible explanation for this could be that higher educational attainments increase the decision-making power of women, and an educated mother might have a better understanding of the benefits of MWHs. However, a study conducted in the Jimma Zone reported that educational status was not associated with MWH use (15). This difference is possibly attributed to the difference in sample size and study design.

The present study also found that the distance from home to the nearest MWH was an independent predictor of the use of an MWH. Women who travel more than 60 min a day are 2.57 times more likely to use an MWH than those who travel for a shorter duration. This finding aligns with those of studies conducted in Zambia, Jimma (Ethiopia), and the Gurage Zone (Ethiopia) (3, 15, 18). The possible explanation for this finding could be that women who travel more will reach a health facility of an MWH almost without any delay because at some point they will already be in the vicinity of a maternity ward and will be guided from there to a health facility or an MWH if the need arises and if they face any serious problems related to delivery. This study also revealed that ANC visits enhance the mother’s use of MWHs. It showed that women who attend ANC follow-ups were 3.5 times more likely to use an MWH than those who did not visit ANC centers at all. This finding is consistent with that of a study done in Zambia (18) and in the Thyolo District of Malawi (24). A possible explanation for this finding is that during ANC visits, healthcare professionals and health extension workers educate and inform pregnant women about the existence of MWHs and advise them to seek voluntary admission to stay in an MWH in their last week of pregnancy, as recommended by the World Health Organization (WHO) (25).

The other most important predictor of MWH use in this study was the experience of complications during past deliveries. Those who suffered complications in previous childbirths were 4.52 times more likely to use MWHs than those who did not. The finding of this study is in line with the studies conducted in Malawi (26), Attat Hospital (Ethiopia) (2), and the Gurage Zone in southern Ethiopia (6). It is possible that complications during previous births make women aware of the dangers of childbirth and cause fear of complications in the current pregnancy. However, another institutional-based study conducted in southern Ethiopia revealed inconsistent findings where previous pregnancy complications were not a significant predictor for MWH utilization (3). This difference may be attributed to the difference in sample size.

This study also revealed that giving birth in a health institution increases the odds of using an MWH. Women who had delivered at a health institution were 6.3 times more likely to use an MWH than those who delivered at home. This is in line with the studies carried out in Zambia (18) and Jinka Zonal Hospital (Ethiopia) (9). Women who delivered in a health facility had higher odds of using MWH facilities. This might be attributed to receiving healthcare provider advice and counsel to stay at an MWH in their final week of pregnancy.

This study also showed that awareness of women on the existence of, as well as the benefit of staying in, an MWH was another important determining factor for MWH use. Women who had awareness were 5.8 times more likely to use MWHs than their non-aware counterparts. A similar finding was observed in another study conducted in the eastern Gurage Zone, Ethiopia, which reported that only 7% of women interviewed were aware of an MWH (6).

The number of children under 5 years old in the household was also found to be an important determinant factor for MWH use. Women who had more than two children under 5 years of age in their household had 88% reduced odds of using an MWH than those who had an equal number of children or less under 5 years of age. This finding is consistent with the empirical findings from other researchers in southern Ethiopia (6, 23). The possible explanation for this is that leaving children at home is not possible and/or does not give comfort to the mother because no one takes care of the children.

### Strengths and limitations of the study

A random selection of the study participants should minimize the likelihood of selection bias. The nature of our study design also allows for looking into multiple determinant factors. Primary outcomes relied on self-reported MWH use by women, which may be subject to recall bias, but the study team made an effort to extract possible misinformation. However, these limitations do not affect the reliability of the findings of the study.

### Conclusion and recommendation

This case–control study sought to determine the factors influencing the use of MWHs in the Gedeo Zone. The results showed that the educational status of women, longer travel time, ANC follow-up, having more than two children under the age of 5 years in the household, experience of complications in previous childbirths, previous place of delivery, and lack of awareness of MWH services were all significantly associated with MWH use. Promoting basic education (especially among women), strengthening the accessibility of transportation for women who decide to stay in an MWH, strengthening and promoting ANC visits, institutional delivery, and counseling clients on the importance and benefits of staying in MWHs are given as recommendations. Finally, these results have general implications for the design, implementation, and scale-up of MWH services in Ethiopia.

## Data Availability

The original contributions presented in the study are included in the article/supplementary material, further inquiries can be directed to the corresponding author.
